# Physical and psychological health consequences of intimate partner violence among married primary school teachers in Delta South Senatorial Zone of Delta State, Nigeria: Implication for marital stability

**DOI:** 10.4314/ahs.v24i1.14

**Published:** 2024-03

**Authors:** Eucharia N Aye, Eze Fidelis Amaeze, Romanus W Aye, Celestine O Eze, Amobi J Onumonu, Chizoba L Obikwelu, Theresa O Oforka

**Affiliations:** Department of Educational Foundations, Faculty of Education, University of Nigeria, Nsukka, Enugu State, Nigeria

**Keywords:** Intimate partner violence (IPV), physical, psychological health consequences, married primary school teachers

## Abstract

**Background:**

Intimate Partner Violence (IPV) is a serious health issue among couples which is recorded more among married partners. Dishearteningly, IPV among couples who are teachers is underreported due to shame, thereby increasing the prevalence of IPV in the area of the study.

**Objectives:**

The study examined physical and psychological health consequences of IPV on married primary school teachers.

**Methods:**

The design was a cross-sectional descriptive survey conducted on married primary school teachers in Delta South Senatorial zone of Delta State, Nigeria from 22nd February - 29th November, 2021. Three hundred and thirteen 313 (207 women and 106 men who have experienced various forms of IPV) who were identified as victims of IPV were used as participants in the study. Structured questionnaire was used to elicit information on physical and psychological health consequences of IPV on married teachers.

**Conclusion:**

The researchers concluded that there are severe and serious physical and psychological health consequences associated with IPV among married primary school teachers in Delta South Senatorial zone of Delta State, Nigeria. Urgent interventions such as public enlightenment, campaigns, workshops, seminars, community health talk-shows should be organized by government stakeholders, non-governmental organizations, community leaders on the prevention of IPV and its dare consequences for marital stability.

## Introduction

Intimate Partner Violence (IPV) is a reproductive and public health challenge and the most frequently committed spousal crimes on women than their male partners. IPV also known as domestic abuse, spousal violence and dating violence refers to any kind of sexual, physical, emotion or psychological violence or abusive behaviours which occurs in an intimate partner relationship between adults or adolescents. IPV is widespread and pervasive in all countries of the world, Nigeria inclusive but it is under reported by victims due to socio-cultural hindrances such as stigmatization and victimization from the members of the public and perpetrators. All over the world, about 1 In 15 women and 1 in 7 men have experienced one of the severe physical violence from an intimate partner in their lifetime; and about 1 in 5 women and 1 in 7 men have experienced contact sexual violence by an intimate partner; and 10% of women and 2% of men reported having been stalked by an intimate partner 4,5. Violence among couples in the home can be linked to domestic violence. Domestic violence also known as IPV is prevalent in the United Kingdom and accounts for 14% of all violent crime and it impacts negatively on health, economic, and social outcomes of families^4^,^6^-^7^.

In Nigeria, a poll report indicated that 28% of Nigerian men and women have experienced either physical or social violence within 12 months of married life^2^. In another study, 30% of women within the age range of 1545 were reported to experience sexual abuse by intimate partner 5. A poll showed that 47% of Nigerians indicated that violence against men by women is in widespread and the spread is as follows: North West 57%, North East 33%, North central 48%, South West 45%, South East 37% and South-South 53% respectively^1^.

IPV can happen to anybody not minding age, race, gender, religion, sexual orientation, educational qualifications, family background and socio-economic status of couples 8,9,11. In South-South Nigeria, IPV of women and men are also spreading like wildfire. It was reported that there are some cases of a man beating, maiming or killing his wife and as well as women dealing with their husbands in like manner^11^

In Delta State, a pregnant mother of eight children was stabbed by her husband to death with a knife as a result of an argument that ensued between them. Research evidences have also shown that IPV is prevalent in Delta State and out of 400 pregnant women in ante-natal clinic of central hospital Ole, a total of 144 (36%) had experienced domestic violence which includes verbal (58%), physical (31%) and sexual (11%) abuse, and their husbands were the offenders but 77% of the pregnant women kept it secret by not reporting to anybody^16^. Another empirical study revealed that men in Delta and Edo states had experienced domestic violence and the reasons behind it ranged from self-defence, provocation, infidelity, financial difficulty and drunkenness^17^. In Delta South zone which is the study area, there are cases of physical and sexual violence against women by their husbands which led to hospitalization of some of the women and death of a third baby due to abandonment by the husband, issues of miscarriages due to battering and finally, divorce ad sharing of children. There are also, cases of physical violence among some couples in the researchers' neighbourhood which led to separations. Similarly, there are husbands who were abused physically and verbally by their wives and they abandoned their homes and wives for their acquaintances where they can have rest. The researchers' personal observation spurred their inquisitiveness to embark on this study.

Equally, literature evidence revealed that women's economic dependence on men as a result of marginalization in man's world, conventional family structure and the treatment of domestic violence as a private matter made the incidence of IPV to be on the increase^17^. More so, IPV is caused by several factors such as patriarchal traditions, cultural and social norms that promote male dominance, power and control over women, gender inequality, discrimination and marginalization of women and lack of self discipline^18^-^20^. Other causes of IPV among couples are poverty, economic or financial dependency, infidelity, childlessness, preference for male child, alcoholism, mental illness, drug abuse, religion, poor anger management, and stress^11^,^21^-^24^. The above factors contributed to incessant marital disharmony, exploitation and marginalization of the most vulnerable group especially the women and girls as well as few men with obvious less power, lack of finance, personality disorders, low level of education and drug abusiveness 21-24.

IPV has grave consequences on the victims. This implies that if IPV remains unchecked, it may cause increase in risk of infectious diseases, reproductive challenges, mental health problems and frequent death. Furthermore, IPV impacts negatively on victims' health (physical and psychological health), social and economic development in particular and the nation's development in general^7^,^10^,^11^. Some of the physical and psychological health consequences of IPV according to empirical evidences are physical inquires (bruises, broken bones, head injuries, back pain, miscarriages, fainting spell and seizures, death, still birth, insomnia, eating disorders, posttraumatic stress disorders, low self-esteem, depression, anxiety and suicidal ideation^21^,^22^,^23^,^24^,^25^. The consequences may not be without some obvious implications on marital stability. Unattended IPV may result into separation, divorce, lack of trust, cheating or death of spouse. It may also imply that marital communication, intimacy and sexual satisfaction may be devoid in the marriage.

Reviewed studies conducted in and outside Nigeria reported that IPV seems to have harmful physical and psychological health effects on women and girls as well as men who are victims 21,22-24,25,28-30. Thus, the reviewed studies were conducted with different subjects such as adolescents, undergraduates, pregnant women and female sex workers but the present study differs from the previous studies' locations. Consequently, no known studies have been conducted on IPV with the addition of male victims depicting dearth of empirical studies. There are evidences on the physical and psychological consequences of IPV on married primary school teachers in Delta State in general and Delta South Senatorial zone in. Moreover, majority of the studies focused on IPV against women and children excluding men who also suffer some forms of physical and sexual violence from their current or former spouses. Therefore, it is against this backdrop that the present study investigated physical and psychological consequences of IPV on couples using a sample of primary school teachers in the study area.

## Methods

Based on the approval by the Department of Educational Foundations, University of Nigeria, Nsukka Ethical Committee for Non-Clinical Research Involving Human Subjects, a cross-sectional descriptive survey statistical analysis was adopted for the study. The study was conducted in Delta State, Nigeria using sample subjects of N=4,156 all married female and male primary school teachers in the eight local government authorities in the Delta South Senatorial zone: Burutu, Bomadi, Patani, Isoko North, Isoko South Warri South, Warri North and Warri South West. The target population was married primary school teachers who have experienced various forms of IPV in the area under study which comprises close to 7.53% of the entire population in the zone. Three hundred and thirteen 313 (207 women and 106 men who have experienced various forms of IPV) identified through preliminary survey participated in the study. Structured questionnaire was used to elicit information on health consequences of IPV from the subjects. Data were collected with the help of five research assistants. The instrument was validated by experts. Field trial testing was used to determine the reliability coefficient of the instrument. The subjects were asked to fill the two clusters of the questionnaire on Physical and Psychological Health Consequences of IPV Questionnaire (PPHCIPVQ) developed by the researchers based on literature evidences.

The PPHCIPVQ has two sections of A and B. Section A elicited information on personal data such as name of school, marital status among others. Section B was divided into two clusters of A and B. Cluster A elicited information on physical consequences of IPV with ten items and cluster B elicited information on psychological consequences of IPV with ten items. All the items were polytomous with response options of strongly agree, agree, disagree and strongly disagree on a four-point rating scale of 4, 3, 2 and 1. The internal consistency reliability coefficient scores of 0.94 and 0.96 were obtained for the two clusters respectively. The reliability scores were considered high for the instrument.

### Data Analysis

The data collected were inputted into SPSS version 18. The data for the research questions were analyzed using descriptive statistics of mean and standard deviation in answering the two research questions. The researchers were able to retrieve 313 copies of the questionnaire. The benchmark of 2.50 of agreeing or disagreeing on an item was arrived at based on the response options of Strongly Agree (4), Agree (3), Disagree (2) and Strongly Disagree (1). Therefore, the decision benchmark is given thus: 4+3+2+1/4 = 10/4 = 2.50.

### Ethical consideration

All the couples (married primary school teachers) signed the researchers' designed consent forms. The researchers obtained ethical approval for the study from the Department of Educational Foundations Research Ethics Committee (REC/EDF/2021/0000112), University of Nigeria, Nsukka.

## Results

Data on table 1 revealed that the primary school teachers comprised 141 Christians (45%), Islam 22(7%), Traditional 91(29%) and Others 60(19%). Years of teaching experience of the married teachers range from (31%), (33%) and (34%). Teachers' years in marriage range from (33%), (33% and (34%). Teachers' educational qualification range from (11%), (34%), (48%) and (55%. Finally, (48%) of married primary school teachers are from urban location while (52%) are from rural areas.

**Table 1:** demographic information of the respondents

Socio-demographic variables of respondents	N (%)
**Religious affiliations**	
Christianity	141(45%)
Islam	22(7%)
Traditional	91(29%)
Others	60(19%)
**Years in teaching**	
5years and below	102(33%)
6years- 10years	97(31%)
11years & above	114(36%)
**Years in marriage**	
5 years & below	107(34%)
6years – 10 years	102(33%)
11 years & above	104(33%)
**Educational qualification**	
Ph.D Degree	35(11%)
Masters Degree	106(34%)
Bachelor Degree	172(55%)
**School location**	
Urban	149(48%)

The physical and psychological health consequences of IPV of the respondents are presented in Table 2. It showed that all the respondents (women and men) strongly agreed and reported that all the items so stated on Table 2 were physical and psychological health consequences of IPV they experienced. The criterion mean scores of women and men participants were 3.52 and 3.50 for physical impacts while psychological impacts were 3.61 and 3.60 respectively.

**Table 2:** Weighted mean and standard deviation on the consequences of IPV

S/n	Health consequences of IPV Physical consequences of PIV	Women		Men		Overall		Decision

		(x)	Std	(x)	Std	(x)	Std	
1.	Acute or traumatic head and face injuries	3.78	0.41	3.80	0.39	3.80	0.39	SA

2.	Insomnia or sleep difficulty	3.85	0.35	3.79	0.40	3.70	0.45	SA

3.	Chronic headaches	3.65	0.35	3.65	0.35	3.65	0.35	SA

4.	chronic pelvic, back and chest pain	3,75	0.44	3.69	0.46	3.70	0.45	SA

5.	Urine and facial incontinence	3.64	0.48	3.70	0.45	3.69	0.46	SA

6.	Loss of appetite or disturbances	2,85	0.38	2.79	0.40	2.80	0.39	SA

7.	Vaginal/sexually transmitted infections	3,21	0.41	3.20	0.40	3.20	0.40	SA

8.	Sexual or reproductive system dysfunction	3.14	0.78	3.12	0.78	0.76	0.76	SA

9.	Miscarriage, unwanted pregnancy or unsafe abortions	3.66	0.49	3.68	0.49	3.67	0.49	SA

10.	Death of victims or still birth	3.60	0.49	3.60	0.49	3.60	0.49	SA

**Aggregate Mean (x)**		**3.52**		**3.50**		**3.49**		

S/N	E-consequences of IPV	Women		Men		Overall		Decision

		(*x̅*)	Std	(*x̅*)	Std_	(*x̅*)	Std	
**11**	Depression and burnout	3.05	0.41	3.50	0.50	3.50	0.50	SA
**12**	Posttraumatic stress disorder	3.92	0.26	3.90	0.30	3.90	0.29	SA
**13**	Anxiety disorder or fear	3.96	0.18	3.95	0.21	3.95	0.21	SA
**14**	Suicidal thoughts or ideations	3.64	0.48	3.65	0.47	3.65	0.47	SA
**15**	Experience of anger and resentment	3.75	0.44	3.74	0.43	3.74	0.43	SA
**16**	Low self-esteem or self-worth or confidence	3.66	0.49	3.68	0.49	3.67	0.49	SA
**17**	Self-blame or self-pity	3.54	0.50	3.52	0.50	3.53	0.50	SA
**18**	Mood swing or feeling of hopelessness	3.35	0.48	3.34	0.47	3.34	0.47	SA
**19**	Inability to trust people	3.42	0.50	3.45	0.49	3.44	0.49	SA
**20**	Nightmares and panic attacks	3.38	0.48	3.34	0.47	3.36	0.49	SA
**Aggregate Mean**		**3.57**		**3.60**	**3.61**		**SA**	
**Key:**	Std- Standard deviation, Mean (*x̅*)							

## Discussion

The present study found that both married primary school women and men agreed that the health consequences associated with IPV are physical and psychological in composition. These findings are supported by the reports that married women and men suffer from physical and Psychological Consequences of ipv^1^,^3^,^5^,^8^,^25^,^26^,^27^. The Physical and psychological consequences of IPV on couples especially women will continue because the victims (women and few men) do not report cases of IPV due to intimidation and fear (on the part of women) from family members, friends and the public. Also, men do not report due to shame and name calling from their follow men. This finding is in line with the earlier studies which reported that physical consequences of IPV among couples were traumatic head and face injuries, insomnia, chronic headaches, STIs, loss of appetite, miscarriages, death among others^21^,^25^,^27^,^29^,^30^. While psychological consequences of IPV among couples based on previous studies were depression, posttraumatic stress disorder, anxiety disorder, suicidal ideations, expression of anger, burnout, low self-esteem, self-blame, nightmares and panic attacks among others^9^,^23^,^24^,^26^,^27^.

The implications of the consequences of IPV among couples especially women are that the victims' lives and homes are shattered and unstable because violence is learned through modeling and imitation and anybody that has been violated in childhood may be prone to violence which may be replicated in the person's later life in marriage. This underpins the fact that there is need for family stability among married primary school teachers for job effectiveness and efficiency as well as self and emotional enhancement. Therefore, there is need to provide counselling services to couples using any cognitive behavioural therapy to restructure the behaviours of the couples for peaceful and smooth relationship. Counselling couples is also very crucial to enable women or men whose lives are being threatened in a sore relationship to leave the union instead of staying there and lose their lives or become permanently disfigured.

## Conclusion

This study affirms that IPV is a serious public health issue which breaches fundamental human rights of women, children and men in the society. IPV is prevalent and pervasive leaving its victims with adverse effect such as traumatic head and face injuries, pains, miscarriage, depression, anxiety, suicidal ideations, burnout, stress, low productivity, huge medical cost for treatment, social distancing, isolation and social displacement. By implication, women or men whose lives are being threatened in a relationship should urgently seek the services of a family counsellor or legal redress to avoid being untimely death. Therefore, an urgent intervention is needed to reduce or curb the prevalence and the grave consequences of IPV on women and few men who are victims by families, communities, health sectors, judiciary, NGOs, and human rights activists using awareness campaign, seeking redress for the victims and implementing the punishment due for perpetrators or offenders to dissuade others from perpetrating the act. The findings of this study will serve as an embodiment of reference materials for future researchers as they will rely on it as an empirical source of information in studies that have similar variables.

### Limitation and strength of the study

The study was unable to look at social consequences of IPV among married primary school teachers in Delta South Senatorial Zone of Delta State. The strength of this study is that it has revealed that married primary school teachers suffer from severe consequences of physical and psychological challenges arising from IPV. The study included married men who are victims of IPV in the zone. The study has also revealed that IPV is common among married primary school teachers in Delta South Senatorial Zone of Delta State.
